# Strategies Typically Developing Writers Use for Translating Thought into the Next Sentence and Evolving Text: Implications for Assessment and Instruction

**DOI:** 10.4236/ojml.2016.64029

**Published:** 2016-08

**Authors:** Jasmin Niedo Jones, Virginia Wise Berninger

**Affiliations:** University of Washington, Seattle, WA, USA

**Keywords:** Cognitive-Linguistic Translation in Typically Developing Writers, Translation Processes for Next Sentence, Translation Processes for Evolving Text, Transcription, Assessment-Instruction Links for Translation Strategies

## Abstract

Three new approaches to writing assessment are introduced. First, strategies for generating the very next sentence are assessed in reference to the local level as well as the evolving text level of composing in progress. Second, strategies for translating thought into written language are coded with transcription (spelling) skill—low, average, or high—held constant. Third, instead of describing composing skill in reference to a single normed score for age or grade in a standardization sample at a static time in development, translation is studied longitudinally when children are in grades 1, 3, and 5 (ages 6, 8, 10) or grades 3, 5, and 7 (ages 8, 10, 12). Applications of the results are discussed for assessment and instruction grounded in levels and generativity of written language and normal variation in typically developing writers.

## 1. Theoretical Framework for Writing Assessment-Instruction Links

### 

#### Cross-disciplinary approach to translation

On the one hand, writing researchers have emphasized the cognitive processes of writing—planning, translating, reviewing, and revising (Hayes & Flower, 1980; Hayes, 2009, 2012a, 2012b), rather than the linguistics or psycholinguistics of writing. On the other hand, linguists have emphasized the generativity of language (from a finite set of words an unlimited number of linguistic structures can be generated) and the deep structures of language underlying the syntax that emerges (Chomsky, 2006) without consideration of the cognitive processes that may be involved.

An interdisciplinary approach is emerging, however, that draws on both the cognitive and linguistic traditions and defining translation as the transformation of thoughts into written language involving bidirectional, cross-do- main cognitive-linguistic communication (Fayol, Alamargot, & Berninger, 2012; Niedo, Abbott, & Berninger, 2014). The cognitive tradition has contributed to understanding that translation into written language may be constrained by transcription abilities, for example, in spelling and/or handwriting. The linguistic tradition has contributed to understanding that written language is both multi-leveled (subword, word, sentence, and text/discourse levels) and generative (Chomsky, 2006). One contribution of the interdisciplinary approach to assessing written language is the increasing evidence that not only syntax is generative (a set of finite items can be used to create an infinite number of constructions in translation into written language, Myhill, 2009) but also so is text at the genre level during the written translation process (Boscolo, Cisotto, & Cortiana, 2014; Olinghouse & Graham, 2009; Olinghouse & Wilson, 2013).

Another contribution of the interdisciplinary approach is the increasing recognition that translation operates differently in conversational registrar of spoken language than in the academic registrar of written language (Silliman & Scott, 2009), even though both draw on different levels of language—word, sentence, and text/discourse levels—but for different purposes. In the case of oral language exchange, the goal is to create cohesion among words within and across language production during turn-taking, which occurs frequently close in time. In the case of written language, the goal is to create coherence among sentences at the genre-constrained discourse level produced across longer time intervals than in conversation by a writer for a non-present audience unlike conversation when the other participant/s are present (see Halliday & Hasan, 1976; Silliman & Scott, 2009).

#### Normal variation-individual differences within and across developing writers

Cross-sectional research has shown that developing writers exhibit intra-individual differences in their relative abilities for writing at the word (spelling and vocabulary), sentence, and text levels (Berninger, Mizokawa, Bragg, Cartwright, & Yates, 1994; Whitaker, Berninger, Johnston, & Swanson, 1994) and use a variety of different text level structures in composing that change across development (Berninger, Fuller, & Whitaker, 1996; Fuller, 1995). However, relatively little research has examined the relationships among strategies used to compose the very next sentence (Level 1 translation) and to compose the evolving overall discourse structure (Level II translation). Most research on self-regulated writing strategies has focused on genre-level writing, for example, for narratives and persuasive essays (for review, see Graham, McKeown, Kiuhara, & Harris, 2012; Graham & Perrin, 2007a, 2007b, 2007c; Harris, Graham, MacArthur, Reid, & Mason, 2011; MacArthur, Graham, & Fitzgerald, 2006; Olinghouse, & Wilson, 2013; Troia, 2009).

Although some research has focused on combining two sentences to express a single idea (Saddler & Graham, 2005) or the nature of idea expression within syntax (Myhill, 2009), relatively little is known, however, about intra- and inter-individual differences for translation for purpose of constructing the very next sentence, which may occur independently of genre, compared to translating at the evolving text level that characterizes specific genres. Yet, struggling writers often are stymied by how to write the very next sentence and sustain the writing process long enough to compose a text of a specific genre.

#### Limitations of current assessment approaches for linking results to instruction

Often normed measures of writing for age or grade peers based on a national standardization sample collected at one point in time are used to assess writing skills. Although research is showing that writing development is a dynamically changing process (Olinghouse & Wilson, 2013; Troia, 2009), normed measures are not available that inform assessment of the normal variation within and among individuals over time. Typically single writing skills are assessed, such as composing ability, without controlling for level of development in other writing skills, such as spelling ability, which may constrain the target writing skills being assessed. Likewise, annual tests yoked to national or state standards that use cut-off points do not provide relevant information about normal variation within a grade or age level or across grade or age levels. Such information is relevant to tailoring instruction to individual differences in developing writers.

## 2. Research Aim

For these reasons, the research aim of the current study was to evaluate whether a coding scheme could be developed that accounted for all observed Level I translating strategies for generating the next sentence and for Level II translating strategies for tying sentences together in the evolving discourse in typically developing writers during elementary and first two grades of middle school (ages 6 to 12). For this research aim, genre was kept constant across specific grade levels in this longitudinal study so that development of translating strategies could be compared for genres kept constant across grade levels as explained in the methods.

## 3. Method

### 3.1. Participants

Through a data sharing plan, compositions were analyzed from a previously published study of developmental change in spelling and related language skills among 60 children who differed as to whether they were reliably good (10 girls, 10 boys), average (10 girls, 10 boys), or poor (10 girls, 10 boys) spellers during a five-year longitudinal study of two cohorts that overlapped in grades three to five: cohort 1 had been assessed annually at the university from grades one to five and cohort 2 had been assessed annually at the university from grades three to seven. The mean ages of children in their first year of the study were 6.8 years for cohort 1 and 8.8 years for cohort 2.

Translation strategies were assessed in narrative composing in grades one, three, and five or in expository in grades two and four in cohort 1; or narrative in grades three, five, and seven or expository in grades four and six in cohort 2. Standard scores for the groups were at or below 95 for poor spellers, between 105 and 112 for average spellers, and 122 or above for good spellers on a nationally normed measure of spelling dictation. For additional details about sample ascertainment, test measures, and research design for studying spelling development and related linguistic skills, see Garcia et al. (2010); that study did not analyze compositions as in the current study and did not examine development of Level 1 or Level 2 Translation Strategies.

Both cohorts were comparable in terms of demographic make-up. In the first cohort, the ethnic identities of children were classified as Caucasian (*n =* 21), Asian-American (*n* = 8), and Native-American (*n* = 1). Their mothers’ educational attainment spanned college (60%), graduate degree (20%), community college/vocational (16.7%), and high school (3.3%). In the second cohort, children’s ethnic identities included Caucasian (*n* = 21), Asian- American (*n* = 7), Black-American (*n* = 1), and other (*n* = 1). Mothers’ educational attainment, which research has shown can be related to their children’s literacy achievement (Benjamin, 1993; McCoy & Cole, 2011), spanned college (40%), graduate degree (40%), community college/vocational (163.3%), high school (3.3%), and unknown (3.3%).

### 3.2. Procedures

In each year of the study, children produced writing in specific genres. Narrative prompts were “*One day at school a surprising or funny thing happened*” (years 1, 3, 5) and “*One weekend at home a funny or surprising thing happened*” (years 3, 5). Expository prompts were “*Explain what a computer is and what it does to someone who has never seen or used one*” and “*Explain what a robot is and can do to someone who has never seen one or used one*” (years 2, 4). Because the nine coded translation strategies did not differ as a function of pen or keyboard production, writing samples were pooled across these modes for this study of translation strategies. However, because of scheduling conflicts and other difficulties five cohort 1 and six cohort 2 children were each missing one writing sample.

The coding units were defined as sentences or main clauses with coordinating clauses (e.g. marked by coordinating conjunctions such as *and*, *but*, *so*, *or*) or subordinate clauses (e.g. marked by subordinate conjunctions such as *after*, *even though*, *although*, *before*, *because*, *before*, *if*). Children’s writings often lacked ending punctuations and sometimes only conjuctions such as *then* or *and* served as markers at the beginning of main clauses. Although a single code often characterized a sentence, in some cases multiple codes applied. For example, consider IA12 *state a wish* and IA16 *If* … *then statement* in this coded next sentence: Even if I can’t have my dream job I hope I can at least have a job that would keep me on my feet.

Two coders developed and practiced applying the coding scheme to 30 writing samples. Each independently coded 10 narrative samples and 10 expository samples. Interrater reliability agreement ranged from .80 to .86 (average .83). The remaining writing samples in the longitudinal sample were then coded independently by both raters and codes compared. Overall, there was 68% agreement across coders for final combined Level I and Level II coding. For the slightly less than a third on which there was disagreement, all were discussed until agreement was reached. In the process it became increasingly apparent that developing writers differed in the extent to which they 1) use more than one translation strategy in generating the very next sentence; and 2) use translation strategies at both Level I and Level II as they write the next sentence. Interrater differences were largely related to coding the use of multiple strategies in parallel. All initial disagreements were readily resolved. See Appendix for final Level I and Level II Coding Schemes that accounted for all observed translation strategies in this study. Overall, all next sentences showed evidence of at least one Level I translation category, but some next sentences showed evidence of use of more than one Level I code or both a Level I and Level II code.

## 4. Results

For this mixed methods study, both descriptive statistics and inferential statistics are reported. The descriptive results specific for grade level and spelling transcription ability indicate which translation strategies students used and can inform instructional strategies that are grade-and genre-yoked that teachers might use in their instructional program for composing. The inferential statistics support generalization of results related to translation strategies across the participants in the current sample.

### 4.1. Descriptive Statistics

Two tables reporting descriptive statistics for frequencies of each coded category in each genre for all participants within a spelling ability group at each grade level are available from the authors. Because of space limitations, only a summary of the main patterns are reported here, first organized by spelling ability and grade levels in which the writing genre was the same (first narrative and then expository). Level 1 translation strategies are presented first followed by Level II translation strategies employing the same organizational structure (spelling ability by grade by genre).

#### Poor spellers

To summarize for *narrative genre*, for Level I translation strategies for the very next written sentences, with the rare exception of one first grader on two occasions, translation resulted in a syntactically complete sentence; and all translation reflected 4 strategies—*stating an opinion or belief*, *describing observable behavior*, *telling the next event*, or *defining what something is*. By third grade, 17 translation categories were observed, but the most frequent were *describing observable behavior* and *telling the next event*. In fifth grade, 18 translation strategies were observed (3 new ones—*telling next step in procedure*, *making a comparison*, and *making an editorial comment*—and 3 used in third grade were not used in fifth grade—*stating a wish*, *a goal/plan*, or *if … then conditions*). Again, in fifth grade, the most frequently used translation strategies were *describe observable behavior* and *tell next event*. In seventh grade, 15 translation strategies were observed, all of which had appeared in earlier grades, except *pose question for reader*. By seventh grade, of the 15 translation strategies observed, all had been used in earlier grades except *pose question for reader*; the most frequently used translation codes included not only *describe observable behavior* or *tell next event*, as in earlier grades, but also *describe by painting a picture with words* or *describing a state of mind or feelings. Indirect dialogue* was also used more frequently than in earlier grades. Overall, the number of Level II translation strategies within and across levels of language increased from first to third grade and thereafter. However, in general, the poor spellers did not tend to apply multiple translation strategies at either Level I or Level II.

To summarize for *expository genre*, in second grade of the 7 translation strategies observed in poor spellers, the most frequent was *state an opinion or belief*. In fourth grade 17 translation strategies were observed. Most frequent were *state an opinion or belief*, *describe observable behavior*, *describe by painting a picture in words*, and *describe function or use*. In sixth grade, 14 translation strategies were observed. Most frequent were *describe function or use*, *state an opinion or belief*, and *describe by painting a picture with words.* The most frequent Level I strategy for within level connections that was used across grade levels was use of pronouns. Few Level II connections across sentences were used for expository genre.

#### Average spellers

To summarize for *narrative genre*, in addition to one presyntactic written language production, average spellers in first grade showed evidence of using 10 Level I translation strategies: *stating an opinion or belief*, *describe by painting picture with words*, *describe state of mind/feelings*, *describe observable behavior*, *tell next event*, *state a goal or plan*, *state an outcome*, *provide an explanation*, *qualify a prior statement*, and *create indirect dialogue*. However, the most frequently used translation category *described an observable behavior*. In addition to these, other translation strategies observed in average spellers in third grade included *state a fact*, *state an opinion or belief*, *tell prior event*, *give example*, *make a comparison*, *make a statement about time or space*, *repeat part of prior text with substitution*, *paraphrase prior text*, *create indirect dialogue*, and *issue direct or indirect command for reader*; but *state a goal or plan*, as had been used by first graders, was not used. At the third grade level, the most frequently used translation categories were *describing by painting a picture with words*, *describing observable behavior*, and *telling next event*. At fifth grade all the translation codes used by third graders, except *give an example*, *make a comparison*, *repeat part of text with substitution*, *create dialogue among characters*, and *issue command for readers*, were observed; but new ones also occurred—*state a goal or plan*, *make a prediction*, *state if … then conditions*, and *make editorial comment for reader*. At fifth grade, the most frequent translation strategies were *describe observable behavior* and *tell next step or procedure*, *qualify a prior statement*, and *describe state of mind or feelings*. At seventh grade, seventeen translation strategies were observed, all of which had been observed in third and/or fifth grade, except *define what something is* and *state a wish*. The most frequent translation strategies in seventh grade included *describe observable behavior* and *tell next event*. Overall, some translation strategies were used frequently across grade levels and some emerged at specific grade levels.

To summarize for *expository genre*, in second grade average spellers used 6 translation strategies. Most frequent were *state opinion or belief* and *describe function or use*. In fourth grade 18 were observed. In fourth grade fourteen translation strategies were observed with the most frequent being *state an opinion or belief*, *describe function or use*, and *describe by painting a picture in words*. In sixth grade 9 translation strategies were observed. Most frequent were *describe function or use*, *state an opinion or belief*, and *describe by painting a picture with words*. As had been the case with poor spellers, the most frequent Level I within-level connections were use of pronouns across grade levels. Overall, average spellers used few Level II connections across sentences for expository genre.

#### Superior spellers

To summarize for *narrative genre*, in first grade 12 translation strategies were observed and no presynaptic written language productions. The most frequently observed translation strategy was *tell the next event*. In third grade 18 translation strategies were observed. Of these, *describe observable behavior* and *tell next event* were most frequent. In fifth grade 22 translation strategies were observed. All had been observed in third grade except *give examples*, *state a wish*, *state if … then conditions*, *pretend or imagine*, and *pose question for reader*. Again the most frequent translation strategies included *describe observable behavior* and *tell next event*. In seventh grade 17 translation strategies were observed of which the most frequent were *describe observable behavior* and *tell next event*. None emerged that had not been observed at an earlier grade. Overall, both Level I and Level II translation strategies were used.

To summarize for *expository genre*, in second grade superior spellers used 13 translation strategies. Most frequent were *state an opinion or belief*, *describe a function or use*, and *describe by painting a picture with words*. In fourth grade they used 15 translation strategies. The same three were most frequent as had been in second grade. In sixth grade they used 11 translation strategies; *describe observable behavior* and *describe state of mind or feelings* were most frequent. Overall, a variety of Level II strategies within and across sentences were observed.

### 4.2. Inferential Statistics

Analyses of variance (ANOVA) were used to identify main effects and interactions with grade and genre for specific coded translation strategies that occurred with some frequency. For *F* and *p* values see Table 1 for cohort 1—narrative, Table 2 for cohort 2—narrative, Table 3 for cohort 1 expository, and Table 4 for cohort 2 expository. The means for significant effects are reported in the text that follows. ANOVAs were followed by post hoc analyses with Bonferroni adjustment comparing change across just two grade levels at a time. Only significant results of those post hoc analyses are reported in the text that follows.

#### Cohort 1—Main effect of grade on Level I translation strategies for narrative genre

Significant developmental changes across grade levels were observed at Level I (write the next sentence) on the following coded translation strategies on which main effects for grade were statistically significant. Frequency of sentences *describing observable behavior* increased from first grade (*M* = .82, *SD* = .48) to third grade (*M* = 1.69, *SD* = .76) to fifth grade (*M* = 1.80, *SD =* 1.06), but was significant only from first to third grade and first grade to fifth grade. Children’s use of *stating the next event* increased from first grade (*M* = .89, *SD* = 1.71) to third grade (*M* = 2.41, *SD* = 2.38) to fifth grade (*M* = 3.43, *SD* = 3.31), but was significant only from first to third grade and first to fifth grade but not from third to fifth grade. *Pretend or imagine what could be* increased from first (*M* = .00, *SD* = .00) to third (*M* = .03, *SD* = .19) to fifth grade (*M* = .27, *SD* = .53), but was not significant from first to third grade, third to fifth grade, or first to fifth grade. Use of *state outcome* increased across the grade levels, from first (*M* = .11, *SD* = .32) to third (*M* = .28, *SD* = .46) to fifth grade (*M* = .47, *SD* = .63) but was significant only from first to fifth grade. Use of *explain* increased from first (*M* = .14, *SD* = .36) to third (*M* = .48, *SD* = .74) to fifth grade (*M* = .53, *SD* = .82), but was significant only from first to fifth grade. Increased use of *qualifying prior statements* was found from first (*M* = .14, *SD* = .45) to third (*M* = .52, *SD* = .83) to fifth grade (*M* = .90, *SD* = 1.13), but was significant only from first to fifth grade.

#### Cohort 1—Main effect of spelling on Level I translation for narrative genre

Better spellers wrote more *tell next event*—poor (*M* = 3.80, *SD* = 4.34), average (*M* = 6.00, *SD* = 3.94), and superior (*M* = 10, *SD* = 3.40) spellers; but only poor and superior spellers differed significantly. Better spellers wrote more *state when and where statements*—poor (*M* = .19, *SD* = .32), average (*M* = .20, *SD =* .42), superior (*M* = 2.00, *SD* = 1.41) spellers; differences were significant between poor and superior spellers and average and superior. Q*ualify prior statement* increased with spelling ability level—poor (*M* = .50, *SD* = .71), average (*M* = 2.00, *SD =* 1.15) and superior (*M* = 2.10, *SD* = 1.85) but only poor and average spellers differed significantly. Superior spellers (*M* = 1.70, *SD* = 1.64) were more likely to use *indirect dialogue* than either average (*M* = .60, *SD* = .52) or poor (*M* = .30, *SD* = .67) spellers, but the groups did not differ significantly from each other.

#### Cohort 1—Interaction of grade and spelling on Level I translation for narrative genre

Only one time-by-spelling ability interaction for *describe by painting a picture in words* was significant in cohort 1, but post hoc tests for the simple effect of group also failed to detect any significant differences between spelling ability at any grade level.

#### Cohort 1—Main effect of grade on Level II translation for narrative genre

*Using pronouns to tie sentences together* increased from grade one (*M* = .47, *SD* = .75) to grade three (*M* = 1.68, *SD* = 2.17) to grade five (*M* = 2.42, *SD* = 2.31), but was significant only for grades one to three and one to five. S*ingle connecting word in independent clause or prepositional phrase to tie words together* increased from grade one (*M* = .28, *SD* = .72) to three (*M* = .73, *SD* = 1.16) to five (*M* = 1.33, *SD* = 1.82), but was significant only between grades one to five. N*arrative genre-characters*—increased from grade one (*M* = .27, *SD* = .53) to three, (*M* = .50, *SD* = .75) to five (*M* = .88, *SD* = .90), but was significant only for grades one to five. *Narrative genre-setting* increased from grade one (*M* = .15, *SD* = .36) to grade three (*M* = .69, *SD* = .72) to grade five (*M* = .75, *SD* = .80), but was significant only between grades one to three and one to five. N*arrative plot introduction statements* increased from grade one (*M* = .18, *SD* = .40) to three (*M* = .68, *SD* = .72) to five (*M* = 1.28, *SD* = .82) and was significant between grades one to three, one to five, and three to five. U*nfolding narrative plot* increased from grade one (*M* = .58, *SD* = 1.82) to three (*M* = 2.81, *SD* = 3.30) to five (*M* = 3.95, *SD* = 3.40), and was significant from grades one to three and one to five. N*arrative plot outcomes* increased from grade one (*M* = .09, *SD* = .19) to three (*M* = .20, *SD* = .40) to five (*M* = .52, *SD* = .64) and was significant between grades one to five (*p* = .001). *Narrative ending statements* increased from grades one (*M* = .15, *SD* = .36) to three (*M* = .52, *SD* = .70) to five (*M* = .81, *SD* = .83), and was significant between grades one to five.

#### Cohort 1—Main effect of spelling on Level II translation strategies for narrative genre

Frequency of applying *pronouns to tie sentences together* was higher for poor (*M* = 2.00, *SD* = 2.36) than average (*M* = 5.60, *SD* = 2.55) spellers (*p* = .084), but more for average than superior (*M* = 5.50, *SD* = 3.87) spellers (*p* = .983), but the difference was significant only for poor and superior spellers. Frequency of u*sing single connecting word in independent clause or prepositional phrase to tie words together* improved with spelling ability—poor (*M* = .80, *SD* = 1.32), average (*M* = 1.70, *SD* = 2.16), and superior (*M* = 4.10, *SD* = 1.66); but the difference was only significant between superior and poor spellers. Narratives of superior spellers (*M* = 2.40, *SD* = 1.78) provided more introduction of characters (*M* = 1.80, *SD* = 1.03) than those for poorer spellers (*M* = .70, *SD* = .95) spellers. Better spellers used setting more in narratives—poor (*M* = .8, *SD* = 1.14), average (*M* = 1.30, *SD* = .95), and superior (*M* = 2.50, *SD* = .97), but the difference was significant only between poor and superior spellers. Better spellers wrote more sentences introducing plot—poor (*M* = .90, *SD* = 1.10), average (*M* = 1.90, *SD* = 1.29), superior (*M* = 3.30, *SD* = .67), but only the difference between superior and poor spellers was statistically significant. Better spellers used more statements about narrative plot in progress (*M* = 2.61, *SD* = 4.77), than average (*M* = 7.10, *SD* = 4.51) or superior (*M* = 11.50, *SD* = 3.72) spellers, but only the difference between poor and superior spellers was significant. Better spellers made *statements about narrative plot outcome* than average (*M* = 1.20, *SD* = .92) and poor (*M* = .20, *SD* = .42) spellers, but the difference was only significant between the poor and average spellers.

#### Cohort 2—Main effect of grade on Level I translation for narrative genre

Results depended on whether the analyses began in grade three rather than grade one and are somewhat different for Level I translation strategies in cohort 2 than for cohort 1. D*escribe a state of mind or feelings* increased from grade three (*M* = .23, *SD* = .51) to five (*M* = .73, *SD* = .78) to seven (*M* = .99. *SD* = 1.06), but was significant only for grade three to seven. *Describe observable behavior* increased from grades three (*M* = 1.44, *SD* = .90) to five (*M* = 2.49, *SD* = 1.42), and then decreased slightly by grade seven (*M* = 2.32, *SD* = 1.05), but the decrease was not significant; however, increased use was significant between grades three to five and three to seven. Frequency for *telling the next event* rose from grade three (*M* = 2.53, *SD* = 2.49) to five (*M* = 3.95, *SD* = 2.91) to seven (*M* = 4.28, *SD* = 3.29); but the difference was significant only for grades three to seven.

#### Cohort 2—Main effect for spelling and interaction with grade on Level I for narrative genre

For this cohort no main effects occurred for spelling ability or interaction for grade-by-spelling ability for Level I.

#### Cohort 2—Main effect for grade on Level II for narrative genre

For the analyses beginning at grade three, rather than grade one, results were also different for Level II translation strategies. N*arrative setting* increased from third (*M* = .50, *SD* = .71) to fifth (*M* = .98, *SD =* .87) grades, but not in seventh grade (*M* = .78, *SD* = .71). No differences were significant. *Using plot introduction* increased from grades three (*M* = .58, *SD* = .50) to five (*M* = 1.13, *SD* = .82) to seven (*M =* 1.32, *SD* = .71) and was significant from grades three to five and from grades three to seven. *Narrative plot outcome* increased from grades three (*M* = .23, *SD* = .43) to five (*M* = .35, *SD* = .56) to seven (*M* = .73, *SD* = .83), but was significant only between grades three and seven.

#### Cohort 2—Main effect for spelling on Level II for narrative genre

*Connecting single word in independent clause or preposition* decreased with increased spelling ability—poor (*M =* 5.50, *SD* = 3.92), average (*M* = 3.70, *SD* = 2.00), and superior (*M* = 1.70, *SD* = 1.25). *Narrative character was used more by* better spellers—poor (*M* = .70, *SD* = .95), average (*M* = 1.10, *SD* = .88), and superior (*M* = 2.20, *SD* = 1.62) spellers; but none of the differences were significant. *Narrative introducing plot* increased with spelling ability—poor (*M* = 1.90, *SD* = 1.45), average (*M* = 3.00, *SD* = 1.41), and superior (*M* = 3.70, *SD* = 1.16); but only poor and superior spellers differed.

#### Cohort 1—Main effect of grade on Level I translation for expository genre

Main effects for grade and significant differences were observed on the following coded translation strategies for writing the next sentence—from grade two (*M =* .36, *SD* = .49) to four (*M* = 1.05 (*SD* = 1.43) in *stating a fact* (*p =* .008); from grade two (*M* = .11, *SD* = .32) to four (*M* = .51, *SD* = 1.04) in *illustrating with examples and/or counter-examples* (*p =* .024); and from grade two (*M* = .00, *SD* = .00) to four (*M* = .46, *SD* = 1.04) in *pretending or imagining what could be*.

#### Cohort 1—Main effect for spelling on Level I translation for expository genre

No main effects for spelling were obtained for Level I translation strategies.

#### Cohort 1—Interaction of grade and spelling on Level I translation for expository genre

There was only one grade-by-spelling ability interaction for *state an opinion*. Post hoc analysis, however, failed to detect a significant difference between spelling groups on use of this coded translation strategy.

#### Cohort 1—Main effect for grade on Level II translation for expository genre

Grade was the only main effect observed for write the next sentence to create a higher-level unfolding discourse structure. Significant main effects were obtained for the following categories: from grades two (*M* = .04, *SD* = .04) to four (*M* = .44, *SD* = .19) on *phrases that repeat prior content*; and from grades two (*M* = .00, *SD* = .00) to four (*M* = .30, *SD* = .71) on *comments that interrupt idea in progress*. Both a main effect for grade and significant grade level differences were observed for the following: between grades two (*M* = .00, *SD* = .00) to four (*M* = .19 (*SD* = .39) on *topic sentence for paragraph*; between grades two (*M* = .00, *SD* = .00) to four (*M* = .33 *SD* = .77) on *compare and contrast organization*; and between grades two (*M* = .00 *SD* = .00) to four (*M* = .33 *SD* = .67) on *ending statements*.

#### Cohort 1—Main effect of spelling and interactions with grade on Level II translation for expository genre

No main effects for spelling ability or interactions with grade were obtained for this translation level.

#### Cohort 2—Main effect of grade on Level I translation for expository genre

There were significant grade effects for *describing a function*, with sixth graders (*M =* 3.46; *SD* = 2.03) applying more of this translation strategy than fourth graders (*M* = 1.68; *SD* = 1.72).

#### Cohort 2—Main effect of spelling on Level I translation for expository genre

Significant effects for spelling ability were found only for the following: *Statements of facts* were more evident in the writings of poor spellers (*M* = 1.40; *SD* = 1.84) than average spellers (*M* = .10, *SD* = .32), and in average than superior spellers (*M* = .00; *SD* = .00). *Pretend or imagine* statements were found more in the writings of superior spellers (*M* = 1.20; *SD* = 1.75) than average spellers (*M* = .10; *SD* = .32), who in turn applied the translation strategy more than poor spellers (*M* = .00; *SD* = .00). *Make a comparison* (*analogy or metaphor*) was used more by those with better spelling—poor (*M* = .10; *SD* = .32), average (*M* = 1.30; *SD* = 1.57), and superior (*M* = .70; *SD* = .95).

#### Cohort 2—Interaction of grade and spelling on Level I translation for expository genre

There was one grade- by-spelling interaction for *makes editorial comments for audience*. However, no significant differences were found between spelling groups at grade four or grade six. Only superior spellers showed significant gains between grades four and six.

#### Cohort 2—Main effect of grade on within Level II translation for expository genre

Significant differences were found between grade levels for use of *topic sentence—*increased use between grades four (*M* = .17; *SD* = .39) to six (*M* = .60; *SD* = .79); for *providing information—*increased used between grades four (*M* = .72; *SD* = 1.88) to grade six, (*M* = 2.27 *SD* = 3.39); and for *ending statement—*decreased between grades four (*M* = .74; *SD* = .38) to six (*M* = .29; *SD* = .46).

#### Cohort 2*—*Main effect of spelling on within Level II translation for expository genre

With better spelling ability, children’s writings evidenced more translation strategies for *connecting words in clauses and phrases—*poor spelling (*M* = 1.10; *SD* = 1.10), average (*M* = .10; *SD* = .32), and superior (*M* = 1.40; *SD* = 1.51); for use of *topic sentence—*poor spelling (*M =* .10; *SD* = .32), average (*M* = 1.00; *SD* = 1.15), and superior (*M* = 1.10; *SD* = .99); for *providing information*—poor spelling (*M* = .20; *SD* = .63), average (*M* = 4.00; *SD* = 4.97), and superior (*M* = 4.30; *SD* = 4.35).

#### Cohort 1—Main Effects across Cohort, Grade, Translation Levels, and Spelling Ability for Narrative

For this Cohort (grades one, three, and five for narrative), there were significant main effects for Grade, *F* (2, 23) = 43.18, *p* < .001, Translation Levels *F* (2, 23) = 51.29, *p* < .001, and Spelling Ability, *F* (2, 24) = 12.92, *p* < .001. Use of all translation strategies improved over time. Overall, Level I (next sentence) translation strategies were used more often than Level II (evolving discourse) translation strategies. In general good spellers outperformed average spellers who outperformed poor spellers. There were no significant interactions.

#### Cohort 1*—*Main Effects across Cohort, Grade, Translation Levels, and Spelling Ability for Expository

For this Cohort (grades two and four for essay) there were two significant main effects and two significant interactions: grade *F* (1, 25) = 41.40, *p* < .001, translation strategies, *F* (1, 25) = 100.50, *p* < .001, translation strategies-by-grade, *F* (1, 25) = 17.37, *p* < .001, and grade-by-spelling, *F* (1, 25) = 4.39, *p* = .023. Overall, the use of each translation code for essay writing increased from second to fourth grade, but in second grade no Level IIB translation strategies (genre-specific) were used. In fourth grade Level IIB translation strategies were used. In second grade, the superior spellers outperformed either the poor or average spellers, but in fourth grade, the superior spellers outperformed the average spellers who outperformed the poor spellers at both Level I (next sentence) and Level II (evolving text).

#### Cohort 2*—*narratives

For this cohort (grades three, five, and seven for narratives), there were main effects for grade, *F* (2, 46) = 4.36, *p* = .015, translation strategy, *F* (2, 46) = 118.90, *p* < .001, a significant interaction for translation strategy-by-spelling ability, *F* (4, 46) = 10.39, *p* < .001 and a significant interaction of grade-by- level, *F* (4, 92) = 3.88, *p* = .006. The interaction of level and grade indicated that Level I and Level IIB (genre-specific) translation strategies increased more than Level IIA (within level cohesion). The interaction with spelling ability indicated that those with greater spelling ability increased their use of level II translation strategies more than those with lower spelling ability. For this cohort (grades four and six for essays), there were main effects for grade, *F* (1, 25) = 7.89, *p* = .01 and translation strategy *F* (2, 25) = 107.85, *p* < .001, and a significant interaction between grade and level of translation strategy, *F* (2, 50) = 3.99, *p* = .025. Overall, across these grades, use of all the translation strategies increased, in general Level I translation strategies were used more often than Level II, but the relative use of Level IIB, compared to Level IIA, increased.

## 5. Discussion

### 5.1. Developmental Changes in Translation Strategies

#### Is cognitive-linguistic translation generative?

A variety of Level I and Level II translation strategies were observed in the narrative and expository compositions, consistent with a generative model of cognitive to linguistic translation (see introduction). However, the nature of the generativity was dynamic, in that the pattern of translation strategies was variable across grade levels, but also constant, in that some comparable translation strategies were used at all or most grade levels. Translation strategies did not develop in a strictly linear fashion.

#### Which translation strategies show developmental change?

In answering this question, keep in mind that translation strategies are not the same as number of sentences written. The next sentence sometimes reflected both a Level I and Level II translation strategy or multiple Level I translation strategies or multiple Level II translation strategies. There were some significant main effects for increased use of a translation strategy across writing development, but specific increases depended on the starting grade level and genre. For Narrative Level I, cohort 1 showed significant developmental increases from grades one to grade five for *describe observable behaviors*, *tell next event*, *pretend or imagine what could be*, *state outcome*, *explain*, *and qualify prior statement*; and cohort 2 showed significant developmental increases from third to seventh grade for *describe a state of mind or feelings*, *describe observable behavior*, and *tell next event.* For expository, cohort 1 showed significant increases from grades two to four on *stating a fact*, *illustrating with examples and/or counter examples*, and *pretending or imaging what could be*; and cohort 2 showed significant developmental change from grades four to six for *describing a function*.

For Level II Narrative cohort 1 increased from grade one to five for *use of pronouns to tie sentences together* and *use of single connecting words* and -*characters*, *-setting*, *-plot introduction*, *-plot in progress*, *-plot outcome*, and *-ending statement*; and for cohort 2, use increased from grade three to seven for *setting*, *introducing the plot*, and *plot outcomes*. For Level II Expository, for cohort 1, use increased from second to fourth grade in *repetition of prior comments* and *comments that interrupt*, *use of a topic sentence for a paragraph*, and genre-specific strategies for *compare and contrast* and *making an ending statement* and for cohort 2, use increased from grades four to six in *topic sentence* and *providing information* but decreased in *ending statements*.

### 5.2. Relationship between Transcription and Translation

#### How are transcription and translation related?

Transcription (spelling ability) is not the same as translation (transforming thoughts into written language). How transcription (spelling*—*may interfere with or support translation may depend on grade levels, genres, and Levels (I and/or II) of translation strategies. Overall, however, the findings varied greatly for different translation strategies, genres, grade, and cohort and showed that translation is a generative process in which thought can be translated into written language in varied ways (cf., Boscolo et al., 2015). Translation of those thoughts into language occurs in multidimensional time—the very next written sentence being constructed in linear time and the unfolding sequence of planning, translating, reviewing, and revising in nonlinear time that ebbs and flows like ocean waves as writers transform their thoughts into written language.

#### What is the nature of translation?

The identified translation strategies in the Appendix are a practical resource for summarizing observed translation strategies typically developing writers used in this process. As such the Appendix provides a reference point from which other researchers can compare translation strategies at Levels I and II observed in the writing samples of the children they study. Much remains to learn about how translation strategies may vary across age levels, genres, and languages. However, the current study shows that while translation may be constrained to some degree by transcription, it is also the case that translation is a separable process from transcription (cf. Alves & Limpo, 2015).

### 5.3. Applications to Educational Practice

#### What are the implications of the current findings for assessment?

The Appendix also provides a useful resource for identifying specific translation strategies at specific grade levels for specific genres during clinical or classroom assessment. Educational and psychological professionals should avoid drawing conclusions about a child’s writing ability based on a single measure of writing—either for composition or for a transcription skill—or writing assessment at a given grade level. On the one hand, written composing is a generative process that draws on multiple levels of language, may change dynamically across grade levels in terms of translation strategies used, and may be constrained due to intra- and inter-individual differences in transcription processes resulting from normal variation. On the other hand, the descriptive analyses provide clues as to what strategies may be most typical at a given grade level for a specific genre for a specific level of spelling (transcription) ability; analyses of such strategies in the writing samples children produce during clinical assessment may help identify those aspects of translation and/or transcription with which particular students struggle and that should be targeted for instruction.

#### What are the implications of the current findings for linking assessment to instruction?

Translation strategies can be taught at Level I—for the very next sentence as well as for both Level I (the very next sentence) and Level II (contribution of the very next sentence to the evolving text of varying genres). Both the descriptive analyses in the text and examples in the Appendix provide insight into which translation strategies might be taught at specific grade levels for specific genre for specific spelling (transcription) abilities.

### 5.4. Limitations and Future Research Direction

Only English speaking children from homes in which the parents were for the most part well educated and highly involved in and supportive of their children’s literacy development participated in this longitudinal study. Future research might extend the research on translation strategies for writing the next sentence to other written languages, cultures, and socioeconomic groups.

Future research should also explore the most effective ways to teach developing writers how to apply both Level I and Level II strategies to their own writing for different genres. Much research on strategy instruction in writing has focused on text-level strategies for specific genres. However in the authors’ teaching, clinical, and research experience, a recurring observation is that many developing writers struggle with what to write next—the very next sentence—and not just genre-specific, text-level composing. Indeed in a related study in which children’s meta-cognitions about writing were studied by interviewing them about how they would explain what writing is to a same-age or younger child, awareness of constructing the very next sentence often emerged prior to or in conjunction with text level composing (Berninger, Gesolowitz, & Wallis, In Press).

In addition, writing instruction would benefit from more research on the complexities of writing deriving from the generativity of written as well as of oral language, the multiple levels of language, normal variation in transcription (spelling) abilities, and variety of cognitive-linguistic translation strategies. Such translation science of research into practice is needed to inform valid assessment-instruction links in an era of both 1) standards-based education and high expectations for all students to meet common standards; and a 2) a knowledge explosion about the biological and environmental diversity of individual written language learners.

## Figures and Tables

**Table 1:** ANOVA results for cohort 1—narrative text (N = 30).

				Partial

	*F*	*df* (time, error)	*p*	η^2^
**Main effect of grade**				

Level I thinking about the next sentence				
Describe observable behavior	14.940	2, 48	.000	.384
Tell next event	7.464	2, 48	.004	.237
Pretend/imagine what could be	4.520	2, 48	.038	.158
State an outcome	4.676	2, 48	.014	.163
Provide an explanation	3.583	2, 48	.035	.130
Qualify a prior statement	4.621	2, 48	.026	.161
Level IIA Connecting sentences—within same level				
Tie sentences with pronoun	7.120	2, 48	.002	.229
Connect with a single word	4.078	2, 48	.042	.145
Level IIB Connecting sentences—across levels (Narrative Genre Organization)				
Character	5.149	2, 48	.009	.177
Setting	7.268	2, 48	.002	.232
Plot introduction	22.633	2, 48	.000	.485
Plot in progress	9.636	2, 48	.000	.286
Plot outcome	8.715	2, 48	.000	.266
Ending statement	6.930	2, 48	.002	.224

**Main effect of spelling**				

Level I thinking about the next sentence				
Tell next event	5.700	2, 24	.009	.322
Make a statement about time/place	12.371	2, 24	.000	.508
Qualify a prior statement	3.449	2, 24	.048	.223
Create indirect dialogue	3.954	2, 24	.033	.248
Level IIA connecting sentences—within same level				
Tie sentences with pronoun	4.412	2, 24	.023	.269
Connect with a single word	7.826	2, 24	.002	.395
Level IIB connecting sentences—across levels (Narrative Genre Organization)				
Character	4.019	2, 24	.031	.251
Setting	6.190	2, 24	.007	.340
Plot introduction	12.060	2, 24	.000	.501
Plot in progress	9.515	2, 24	.001	.442
Plot outcome	4.295	2, 24	.025	.264

**Interaction grade × spelling**				

Describe—paint pictures with words	3.816	4, 48	.015	.241

**Table 2:** ANOVA results for cohort 2—narrative text (N = 30).

				Partial

	*F*	*df* (time, error)	*p*	η^2^
**Main effect of grade**				

Level I thinking about the next sentence				
Describe state of mind/feelings	5.191	2, 46	.009	.184
Describe observable behavior	7.131	2, 46	.002	.237
Tell next event	3.317	2, 46	.045	.126
Level IIB connecting sentences—across levels (Narrative Genre Organization)				
Setting	3.879	2, 46	.042	.144
Plot introduction	9.797	2, 46	.000	.299
Plot outcome	5.354	2, 46	.016	.189

**Main effect of spelling**				

Level IIA connecting sentences—within same level				
Connect with a single word	3.773	2, 23	.038	.247
Level IIB connecting sentences—across levels (Narrative Genre Organization)				
Character	4.637	2, 23	.020	.287
Plot introduction	4.588	2, 23	.021	.285

**Interaction grade × spelling**				

State an outcome	2.972	4, 46	.029	.205
State an ending	2.865	4, 46	.034	.199

**Table 3:** ANOVA results for cohort 1—expository text (N = 30).

				Partial

	*F*	*df* (time, error)	*p*	η^2^
**Main effect of grade**				

*Level I thinking about the next sentence*				
State a fact(s)	8.399	1, 25	.008	.251
Illustrate—example/counter-example	5.751	1, 25	.024	.187
Pretend/imagine what could be	5.171	1, 25	.032	.171
*Level IIA connecting sentences*—*within same level*				
Repeat/construct prior proposition/content	4.420	1, 25	.046	.150
Interrupt idea in progress	5.305	1, 25	.030	.175
*Level IIB connecting sentences*—*across levels* (*Narrative Genre Organization*)				
Topic sentence for paragraph	6.735	1, 25	.016	.212
Compare and contrast	5.918	1, 25	.022	.191
Ending Statement	6.519	1, 25	.017	.207

**Interaction grade × spelling**				

State an opinion or belief	3.803	2, 25	.036	.233

**Table 4:** ANOVA results for cohort 2—expository text (N = 30).

				Partial

	*F*	*df* (time, error)	*p*	η^2^
**Main effect of grade**				

*Level I thinking about the next sentence*				
Describe function or use	25.226	1, 25	.000	.502
*Level IIB connecting sentences*—*across levels* (*Narrative Genre Organization*)				
Topic sentence for paragraph	9.461	1, 25	.005	.275
Provide information	5.912	1, 25	.023	.191
Ending statement	4.755	1, 25	.039	.160

**Main effect of spelling**				

*Level I thinking about the next sentence*				
State a fact(s)	4.777	2, 25	.018	.276
Pretend/imagine what could be	3.719	2, 25	.039	.229
Make a comparison—analogy/metaphor	3.440	2, 25	.048	.216
*Level IIA connecting sentences*—*within same level*				
Connect with a single word	3.652	2, 25	.041	.226
*Level IIB connecting sentences*—*across levels* (*Narrative Genre Organization*)				
Topic sentence for paragraph	3.649	2, 25	.041	.226
Provide information	3.476	2, 25	.047	.218

**Interaction grade × spelling**				

Make editorial comment for reader	4.474	2, 25	.022	.264

## Appendix: Coding Scheme for Translation Strategies and Examples

### I. Coding Thinking about Writing the Next Sentence

#### A. Add Information

IA1. State fact or facts for which there is general agreement.
  Narrative: The dog was a great Dane named Tiny.
  Essay: Robts have a lot of tenolagy in them.
IA2. State an opinion (belief).
  Narrative: It was reeeeeeeeaaaaaaaaaaaaaly funny.
  Essay: playing on the computer is the funniest.
IA3. Describe-paint a picture with words.
  Narrative: Its face was made of mashed potatoes and its eyes were of chocolate pudding.
  Essay: A computer is a machine that has a screen.
IA4. Describe a state of mind or feelings.
  Narrative: When I finally got in the hang of things I was filled with joy!
IA5. Describe a function or use of an object.
  Essay: A computer is something that people use to write letters or emails.
IA6. Describe observable behavior.
  Narrative: It jumped down and went away as I started to get up.
IA7. Tell the next step or procedure. (Note this is also if … then implied).
  Essay: so if anyone calls you while you’re on the internet, they will get the busy signal, and have to try to call again.
IA8a. Tell next event.
  Narrative: *Someone started a food fight.* He got detention for 10 days.
IA8b. Tell prior event.
  Narrative: *Someone started a food fight. He got detention for* 10 *days. He wasn’t allowed to talk in the school for those ten days.* The food fight lasted for an hour and 40 minutes.
IA9. Define what something is.
  Narrative: In French, conge means “day off”.
  Essay: Email is an online postcard you can send to different people.
IA10. Define what something is not.
  Essay: *You can do a lot of things on a computer like panit* [*paint*]. Not pad (you can write on that).
IA11. Illustrate-give one or more examples or counter-examples.
  Essay: On your computer you can also play games like pinball minds wipper, salter [solitary], chackers.
IA12. State a wish.
  Narrative: I hope that this year it snows again so that I can re-live the memory with my friends.
  Essay: sometimes I wish I had a robot so it could help me with my home work.
IA13. State a goal/plan.
  Narrative: at the end of recess we were going to open it.
IA14. Tell a plan for reaching the goal.
  Narrative: will ask teacher to help open it.
IA15. Make a prediction.
  Essay: Someday in the future, I think computers might be like a friend.
IA16. State conditions If … then … (then may be implied not stated).
  Essay: and if you pay a carten [certain] amot of money ever month you can have the intrat [internet].
IA17. Pretend or imagine what could be but does not necessarily exist.
  Narrative: An elephant sitting on a kangaroo flew into school on a blueprint for a U.F.O.!
  Essay: A robot can entertain you with a po up t.v.
IA18. Make a comparison (analogy or metaphor).
  Narrative: and then I knocked over every one in my line like dominos.
  Essay: And e-mail is like sending letters to your friends on the computer.
IA19. State an outcome.
  Narrative: She flew us home, and that was the end of that.
IA20. Make a statement about time (when) and space (where).
  Narrative: I woke up on a Sunday morning in December and my roof was white!

#### B. Provide an explanation

Narrative: The surprising thing that happened was Spenser was not in my class.
  Essay: The best part in using a computer is the internet because you can find games and information.

#### C. Modify Text

IC1. Qualify a prior statement—place limit(s) on it.
  Narrative: *I was standing in front of my locker when I heard a strange noise.* It was coming from an empty class room.
  Essay: *There are few actual robots in the real world I believe.* There are a few of what you typically think of as a robot at least.
IC2. Evaluate content or organization of what you are writing.
  Essay: This is part of reasons.
IC3. Repeat part of prior text with substitution.
  Narrative: *It was surprising because I thought I wouldn’t get that in the third grade.* That’s the surprising thing.
IC4. Paraphrase prior text.
  Essay: Robots are a type of machine. They are used for many different things. Some robots are toys and are used to entertain while others are used for different things like helping do everyday things. Robots are made of metal, and can often have similar features to humans. *They are useful and can be made and work in many different ways.*

#### D. Create Dialogue

ID1a. Direct dialogue among characters.
  Narrative: My brother’s pet guinea pig started yelling, “I’m a rebel guinea squirrel!” over and over again.
ID1b. Indirect dialogue among characters.
  Narrative: She said that my teacher was gone and she was the substitute.
ID2a. Pose question for reader audience.
  Narrative: The end or is it?
  Essay: *Then, robots would be a species.* Wouldn’t that be cool?
ID2b. Make editorial comment for the reader audience.
  Narrative: I bet that was a good way for him to start off his day.
  Essay: You’ll find that it [*computer*] is really fun.
ID2c. Issue direct or indirect command for reader audience.
  Narrative: I don’t want you to laugh ok.
  Essay: You should take lessons from someone like me on how to use a computer.

### II. Coding Connecting Sentences Together

#### A. Within Same Level

IIA1a. Tie sentences with a pronoun.
  Narrative: *I was on my way to class when my friend Nigel slipped past me a fell into a garbage can.* I laughed and asked him was the rush?
  Essay: *a computer is a machine that runs on batteries/*it is like a imfermation gatherer.
IIA1b. Connect sentences with a word in subordinate clause.
  Narrative: *It took about* 50 *minutes to install.* When it was done installing, my friend Dylan came over.
IIA1c. Connect sentences with a single word (e.g. *then* at beginning of independent clause or *after* in prepositional phrase).
  Narrative: *I kind of thought it was power out.* Then, I saw how heavy the rain was.
  Essay: You can also watch movies on it [*computer*].
IIA1d. Phrase that repeats or constructs prior proposition or content.
  Essay: *A robot could be made out of almost anything.* Metal is the main thing people like to make robots out of.
IIA2. Make a comment that interrupts the idea in progress and then continues with that idea unless at end because time runs out.
  Essay: *The robot could be nice and try to serve them*, *but they might think a drink would be poison. They might think that food would have injected poison.* A robot could be made out of almost anything. Metal is the main thing people like to make robots out of….

#### B. Across Levels

IIB1a. Narrative Genre Organization: Characters.
IIB1b. Narrative Genre Organization: Setting (place and/or time).
IIB1c1. Narrative Genre Organization: Plot introduction or.
IIB1c2. Narrative Genre Organization: Plot in progress.
IIB1d. Narrative Genre Organization: Plot outcome.
IIB1e. Narrative Genre Organization: Ending statement.
I was in bed at about ten o’clock when I woke up. IA20; IIB1b, IIB1c1.
I heard a noise that sounded like my cat. IA6; IIB1c2.
I started to look down over my bunk bed but couldn’t see anything. IA8a; IIB1c2.
I lied back down when suddenly a cat (not mine) jumped up onto my bunk bed and hissed at me. IA8a; IIB1c2.
I remembered that we had been leaving the back door open so our cats could do there business outside. IA8a; IIB1c2.
That must have been how it got in. IB; IIA1a.
It jumped down and went away as I started to get up. IA8a; IIA1a; IIB1e.
It was reeeeeeeeaaaaaaaaaaaaaly funny. IA2; IIA1a.
THIS IS A TRUE STORY! ID2b; IIA1a, IIB1e.
IIB2a. Expository Genre Organization: Topic sentence for paragraph.
IIB2b. Expository Genre Organization: Provide information.
IIB2c. Expository Genre Organization: Compare and contrast.

Robots have different feet. Some robots haven’t.

IIB2d. Expository Genre Organization: Take position and defend (persuasive).
IIB2e. Expository Genre Organization: Summary so far.
IIB2f. Expository Genre Organization: Conclusion.
IIB2g. Expository Genre Organization: Ending statement.
Computers come in many shapes and sizes. IA3; IIB2a.
They are usually they are shaped like boxes, but some computers are flat screeened. IA3, IA11; IIA1a, IIB2b.
The screen is black when the computer is not being used. IA3; IIB2b
The outside of the computer is probably grey or black. IA3; IIB2b.
On the computer you can do many things. IA5; IIA2; IIB2a.
You can write with the keyboard. IA5; IIB2b.
You can also make graphs and charts. IA5; IIB2b.
Finally, computers are used a lot for math. IA5; IIA1c, IIB2g.
